# Supplementary material for: Distributed optimal power flow
Source: PLoS One. 2021 Jun 18;16(6):e0251948. doi: 10.1371/journal.pone.0251948 (PMC8213183; doi:10.1371/journal.pone.0251948)
Supplement: S1 Appendix — (DOCX) [file pone.0251948.s001.docx]

**S1 Appendix. Proof of claims**

### **Claim 1**: The matrices associated with power flows and power injections are all of rank 4.

**Proof**: The power flows and injections are the product between the voltages and current complex conjugate, ***vi****^*^*. The power at the *i*^th^ node is:$p_{i}+jq_{i}=v_{i}i_{i}^{*}=v^{T}S_{i}v=v^{T}\left(\breve{S}_{i}+j\bar{S}_{i}\right)v$. Let the real-valued matrix sandwiched by *v* be *M_i_*. For *M_i_* has two nonzero rows at *i* and *Nb+i* rows, $M_{i}=2e_{i}a_{i}^{T}+2e_{i+Nb}b_{i}^{T}$ where *a_i_* and *b_i_* are column vectors. Because *p_i_* and *q_i_* are scalar, the real-valued can be replaced by symmetric $\left[M_{i}\right]=\frac{1}{2}\left(M_{i}+M_{i}^{T}\right)$. The matrix [*M_i_*] is $\left[M_{i}\right]=e_{i}a_{i}^{T}+a_{i}e_{i}^{T}+e_{i+Nb}b_{i}^{T}+b_{i}e_{i+Nb}^{T}$. The eigenvalue decomposition of the first pair of components leads to $e_{j}a_{j}^{T}+a_{j}e_{j}^{T}=\lambda_{1}u_{1}u_{1}^{T}+\lambda_{2}u_{2}u_{2}^{T}$ where $\lambda_{1}=\frac{1}{2}\left\|e_{j}+a_{j}\right\|^{2},$ $u_{1}=\frac{e_{j}+a_{j}}{\left\|e_{j}+a_{j}\right\|}$, $\lambda_{2}=-\frac{1}{2}\left\|e_{j}-a_{j}\right\|^{2}$, and $u_{2}=\frac{e_{j}-a_{j}}{\left\|e_{j}-a_{j}\right\|}$. The dimension of the null space of the matrices is (*2nb – 2*). According to the rank-nullity theorem, the rank of the first pair is *2nb*  – (*2nb – 2*) = 2. Similarly, the rank of the last pair of two components also reveals a rank 2 decomposition. Because *M_i_* is a full row-rank matrix, the first and last pairs of components are independent (i.e., each pair spans the real and imaginary voltage spaces); hence, they do not overlap. Therefore, [*M_i_*] is a rank 4 matrix. The proof for the ranks of the matrices associated with the power flows follows the same steps.

**Claim 2**: The matrices associated with the squares of the voltage magnitudes have rank 2.

**Proof**: The voltage magnitude squared at Node *i* is $E_{j}=v_{xi}^{2}+v_{yi}^{2}$ $=v^{T}\left(e_{i}e_{i}^{T}+e_{i+Nb}e_{i+Nb}^{T}\right)v$. The eigenvector decomposition of the matrix sandwiched by the voltages reveals the rank-2 matrix.
