# Supplementary material for: Distributed optimal power flow
Source: PLoS One. 2021 Jun 18;16(6):e0251948. doi: 10.1371/journal.pone.0251948 (PMC8213183; doi:10.1371/journal.pone.0251948)
Supplement: S2 Appendix — (DOCX) [file pone.0251948.s002.docx]

**S2 Appendix. Proof of convergence**

**Strategy of the proof**

The proof comprises of three parts. The first part reveals the relationship between the local variables and the central variable at each iteration and proves that the local and central variables are bounded by the nodal updates. It also shows that the surrogate function always improves at each iteration. The second part shows that the surrogate function converges to a local minimum. The third part proves that the original nodal distributed OPF and the surrogate function share the same fixed point. This concludes the convergence of the proposed algorithm.

The nodal OPF problem was presented. The generalized OPF problem *w* is described in Eq. (1) below:

$\min_{x_{j}\in X_{j}} \sum_{j} w_{j}\left(x_{j},y,z_{j}\right)=\min_{x_{j}\in X_{j}} \sum_{j} \left[f_{j}\left(x_{j}\right)+g_{j}\left(x_{j},y,z_{j}\right)\right]$ (A1)

where *x* is the nodal primal variable, $y=\left(\begin{matrix} v_{L}^{T} & v_{M}^{T} \end{matrix}\right)^{T}$, *z* represents the multipliers, *Ф_j_* is the collection of $\Phi_{j}^{M_{eq}}$ and $\Phi_{j}^{M_{in}}$corresponding to all the constraints for the *j*^th^ nodal OPF, and *X_j_* is the column space of *Ф*_j_ at *j*.

Eq. (A1) implies that:

- ***w_j_*** is coercive.
- ***f_j_*** is nonconvex, where , and has the following properties: 1) *f_j_* is a nonconvex but smooth function that is *C^1^* on an open set containing *X_j_* (defined by the column space of *Ф_j_*), and 2) $\nabla f_{j}$ is Lipschitz continuous on *X_j_* where each *X_j_* is nonempty, closed, and convex.
- ***g_j_*** is a quadratic ADMM function defined as $g\left(x_{j},y,z_{j}\right)=\frac{\rho_{j}}{2}\left\|x_{j}-\Phi_{j}^{T}y\right\|^{2}+z_{j}^{T}\left(x_{j}-\Phi_{j}^{T}y\right)$.

Even though the decision variables in (1) have low cardinality, the uniqueness and the existence of the solution are not guaranteed due to the nonconvex nature of the problem. To address the complexity issue, a surrogate function *h_j_* is introduced:

$H\left(x,y,z\right)=\sum_{j} h_{j}\left(x_{j},y,z_{j}\right)=\sum_{j} \left[u_{j}\left(x_{j}\right)+g_{j}\left(x_{j},y,z_{j}\right)\right]$ (2) where $u_{j}\left(x_{j}\right)=\text{SDP of }f_{j}\left(x_{j}\right)$ (A2)

Because *u_j_* is the SDP relaxation of *f_j_*, it is: 1) strongly convex, 2) Lipchitz continuous, 3) continuously differentiable on *X_j_*. and 4) $\nabla_{x_{j}}f_{j}\left(x_{j}\right)=\nabla_{x_{j}}u_{j}\left(x_{j}\right)$.

**Part I: Boundedness of the updates of the variables**

The ADMM-type distributed algorithm comprises four update processes – *x*- (Rule 3) and *y*-optimizations (Rule 5), as well as *z*- (Rule 5) and *x*-updates (Rule 6).

## **Rule 3: x-optimization**

For the solution $\tilde{x}_{j}^{k+1}$ found in Rule 3, where , the optimality condition yields:

$\nabla_{x_{j}}u_{j}\left(\tilde{x}_{j}^{k+1}\right)+z_{j}^{k}+\rho_{j}\left(\tilde{x}_{j}^{k+1}-x_{j}^{k}\right)=0$ (A3)

or

$\nabla_{x}U\left(\tilde{x}^{k+1}\right)+z^{k}+D_{\rho}\left(\tilde{x}^{k+1}-x^{k}\right)=0$ in the vector form (A4)

where and . We also define , the (*k+1*)^th^ update , and . The update in the *x*-variable is generalized as $\hat{x}_{j}^{k+1}-x_{j}^{k}=\left(1-\delta_{j3}\right)\Delta^{k}\left(\tilde{x}_{j}^{k+1}-x_{j}^{k}\right)+\delta_{j2}\Delta^{k}\tilde{\varepsilon}_{j}^{k+1}$, where $\delta_{jm}$ is the Kronecker delta that equals 1 for *j = m* (i.e., $j\in\Omega_{m}$) and 0 otherwise. Rule 4 leads to:

$\hat{\zeta}^{k+1}=x^{k}+\Phi^{T}\left(\Phi D_{\rho}\Phi^{T}\right)^{-1}\Phi D_{\rho}\left(\tilde{x}^{k+1}-x^{k}\right)_{\Omega_{3}^{c}}$ (A5)

and

$\hat{x}^{k+1}=x^{k}+\Delta^{k}\left(\tilde{x}^{k+1}-x^{k}\right)_{\Omega_{3}^{c}}+\Delta^{k}\left(\tilde{\varepsilon}^{k+1}\right)_{\Omega_{2}}$ where and (A6)

## **Rule 5: y-optimization and z-update process**

*y^k+1^* satisfies the optimality condition $\nabla_{y}G\left(\hat{x}^{k+1},y^{k+1},z^{k}\right)=-\Phi D_{\rho}\hat{x}^{k+1}+\Phi D_{\rho}\Phi^{T}y^{k+1}-\Phi z^{k}=0$ in the *y*-optimization, where and . The solution is $y^{k+1}=\left(\Phi D_{\rho}\Phi^{T}\right)^{-1}\left(\Phi D_{\rho}\hat{x}^{k+1}+\Phi z^{k}\right)$. Using the solution and the *z*-update in Rule 5, $z^{k+1}=D_{\rho}\hat{x}^{k+1}-D_{\rho}\Phi^{T}y^{k+1}+z^{k}$, it is found that $z^{k+1}=\left[I-D_{\rho}\Phi^{T}\left(\Phi D_{\rho}\Phi^{T}\right)^{-1}\Phi\right]\left(D_{\rho}\hat{x}^{k+1}+z^{k}\right)$. Multiplication with the matrix Ф yields $\Phi z^{k+1}=0$ for all *k*.

$\Delta z^{k+1}=z^{k+1}-z^{k}=\left[I-D_{\rho}\Phi^{T}\left(\Phi D_{\rho}\Phi^{T}\right)^{-1}\Phi\right]D_{\rho}\hat{x}^{k+1}$ (A7)

$y^{k+1}=\left(\Phi D_{\rho}\Phi^{T}\right)^{-1}\Phi D_{\rho}\hat{x}^{k+1}$ (A8)

Even though the cardinality of *y* increases with the system size, its computation in (A8) is the linear combination of nodal updates. Note that $\left(\Phi D_{\rho}\Phi^{T}\right)^{-1}\Phi D_{\rho}$ does not change with the iteration and that with each column in $\left(\Phi D_{\rho}\Phi^{T}\right)^{-1}\Phi D_{\rho}$, the nodal variable $\hat{x}^{k+1}$ finds a corresponding *y*-variable, i.e., $y^{k+1}=\sum_{j} y_{j}^{k+1}$where$y_{j}^{k+1}=\left(\Phi D_{\rho}\Phi^{T}\right)^{-1}\Phi D_{\rho}e_{j}\hat{x}_{j}^{k+1}$. Similarly, $z^{k+1}=z^{k}+\Delta z^{k+1}=z^{k}+\sum_{j} \Delta\hat{z}_{j}^{k+1}$where$\Delta\hat{z}_{j}^{k+1}=\left[I-D_{\rho}\Phi^{T}\left(\Phi D_{\rho}\Phi^{T}\right)^{-1}\Phi\right]D_{\rho}e_{j}\hat{x}_{j}^{k+1}$. It is possible to compute $y_{j}^{k+1}$ and $\Delta\hat{z}_{j}^{k+1}$ at each node without an information exchange with any other nodes. While the *y*-optimization and the *z*-update process involve central information exchanges, the computations are linear updates of nodal $y_{j}^{k+1}$ and $\Delta\hat{z}_{j}^{k+1}$.

## **Rule 6: x-update**

According to Rule 6, *x* is the projection of $\hat{x}^{k+1}$ on *X*, i.e., $x^{k+1}=\Phi^{T}\left(\Phi D_{\rho}\Phi^{T}\right)^{-1}\Phi D_{\rho}\hat{x}^{k+1}$, which yields:

$\Phi D_{\rho}x^{k+1}=\Phi D_{\rho}\hat{x}^{k+1}$ (A9)

The projection on the real space of Ф is done to make the $\hat{x}^{k+1}$ variable internally consistent with the global variable *y*, $x^{k+1}=\Phi^{T}y^{k+1}$. Since *x^k^* is strictly in the column space of *Ф*, $x^{k}=\Phi^{T}\left(\Phi D_{\rho}\Phi^{T}\right)^{-1}\Phi D_{\rho}x^{k}$, i.e., the linear projection of a vector onto its full space is the vector itself, and

$x^{k+1}-x^{k}=\Phi^{T}\left(\Phi D_{\rho}\Phi^{T}\right)^{-1}\Phi D_{\rho}\left(\hat{x}^{k+1}-x^{k}\right)$ (A10)

$y^{k+1}-y^{k}=\left(\Phi D_{\rho}\Phi^{T}\right)^{-1}\Phi D_{\rho}\left(x^{k+1}-x^{k}\right)$ (A11)

$z^{k+1}-z^{k}=\left[I-D_{\rho}\Phi^{T}\left(\Phi D_{\rho}\Phi^{T}\right)^{-1}\Phi\right]D_{\rho}\left(\hat{x}^{k+1}-x^{k}\right)$ (A12)

Eq. (A10) indicates that a linear mapping exists between $x^{k+1}-x^{k}$ and $\hat{x}^{k+1}-x^{k}$. Eq. (A12) implies that $z^{k+1}-z^{k}\in\text{Imag}\left[I-D_{\rho}\Phi^{T}\left(\Phi D_{\rho}\Phi^{T}\right)^{-1}\Phi\right]$. Note that $\left[I-D_{\rho}^{1/2}\Phi^{T}\left(\Phi D_{\rho}\Phi^{T}\right)^{-1}\Phi D_{\rho}^{1/2}\right]D_{\rho}^{1/2}\Phi^{T}=0$, i.e., $\Phi D_{\rho}^{1/2}$ exists in the null space of$\left[I-D_{\rho}^{1/2}\Phi^{T}\left(\Phi D_{\rho}\Phi^{T}\right)^{-1}\Phi D_{\rho}^{1/2}\right]$. According to Rule 6, i.e., $x^{k}=\Phi^{T}y^{k}$ for all *k*, $\Delta x^{k+1}$ is also in the column space of Ф. Then, $\left[I-D_{\rho}^{1/2}\Phi^{T}\left(\Phi D_{\rho}\Phi^{T}\right)^{-1}\Phi D_{\rho}^{1/2}\right]D_{\rho}^{1/2}\Delta x^{k+1}=0$. From Eq. (A7), we arrive at $\left(\Delta z^{k+1}\right)^{T}D_{\rho}^{1/2}\Delta x^{k+1}=0$, meaning that the update of the primal variables and the multiplier update are perpendicular. Hence: $D_{\rho}^{1/2}\Delta x^{k+1}=D_{\rho}^{1/2}\Phi^{T}\left(\Phi D_{\rho}\Phi^{T}\right)^{-1}\Phi D_{\rho}^{1/2}D_{\rho}^{1/2}\Delta x^{k+1}$. The singular value decomposition of $\Phi D_{\rho}^{1/2}$, i.e., $\Phi D_{\rho}^{1/2}=U\Sigma V^{T}$, yields: $D_{\rho}^{1/2}\Delta x^{k+1}=V\left[\begin{matrix} \Sigma_{R} \\ 0 \end{matrix}\right]U^{T}\left(U\Sigma_{R}^{2}U^{T}\right)^{-1}U\left[\begin{matrix} \Sigma_{R} & 0 \end{matrix}\right]V^{T}D_{\rho}^{1/2}\Delta x^{k+1}$ which is further simplified $D_{\rho}^{1/2}\Delta x^{k+1}=V_{R}V_{R}^{T}D_{\rho}^{1/2}\Delta x^{k+1}$. Therefore,

$V^{T}D_{\rho}^{1/2}\left(x^{k+1}-x^{k}\right)=V_{R}^{T}D_{\rho}^{1/2}\left(x^{k+1}-x^{k}\right)$ (A13)

Eq. (A13) means that $D_{\rho}^{1/2}\left(x^{k+1}-x^{k}\right)$ stays strictly in the real space of *V* or of $\Phi D_{\rho}^{1/2}$.

On the other hand, from Eq (A10), it can be seen that$\Phi D_{\rho}^{1/2}\left[D_{\rho}^{1/2}\left(x^{k+1}-\hat{x}^{k+1}\right)\right]=0$, i.e., $D_{\rho}^{1/2}\left(x^{k+1}-\hat{x}^{k+1}\right)$ stays strictly in the null space of *V* or of $\Phi D_{\rho}^{1/2}$. Using Eq. (A13), it can be seen that $D_{\rho}^{1/2}\left(x^{k+1}-x^{k}\right)$ and $D_{\rho}^{1/2}\left(x^{k+1}-\hat{x}^{k+1}\right)$ are perpendicular. According to (A6),

$\left\|\hat{x}^{k+1}-x^{k}\right\|=\Delta^{k}\left\|\left(\tilde{x}^{k+1}-x^{k}\right)_{\Omega_{3}^{c}}+\left(\tilde{\varepsilon}^{k+1}\right)_{\Omega_{2}}\right\|\leq\Delta^{k}\left\|\tilde{x}^{k+1}-x^{k}\right\|_{\Omega_{3}^{c}}+\Delta^{k}\left\|\tilde{\varepsilon}^{k+1}\right\|_{\Omega_{2}}\leq\Delta^{k}\left(1+\tau_{\max}^{k}\right)\left\|\tilde{x}^{k+1}-x^{k}\right\|_{\Omega_{3}^{c}}$ (A14)

Eqs. (A10), (A11), (A12), and (A14) yield:

$\left\|x^{k+1}-x^{k}\right\|=\left\|D_{\rho}^{-1/2}V\Sigma^{T}\Sigma_{R}^{-2}\Sigma V^{T}D_{\rho}^{1/2}\left(\hat{x}^{k+1}-x^{k}\right)\right\|\leq\Delta^{k}\left(1+\tau_{\max}^{k}\right)\sqrt{\frac{\rho_{\max}}{\rho_{\min}}}\left\|\tilde{x}^{k+1}-x^{k}\right\|_{\Omega_{3}^{c}}$ (A15)

$\left\|y^{k+1}-y^{k}\right\|=\left\|U\left[\begin{matrix} \Sigma_{R}^{-1} & 0 \end{matrix}\right]V^{T}D_{\rho}^{1/2}\left(x^{k+1}-x^{k}\right)\right\|\leq\frac{\Delta^{k}\left(1+\tau_{\max}^{k}\right)\sqrt{\rho_{\max}}}{\left|\sigma_{\Phi D_{\rho}^{1/2}}^{\min}\right|}\left\|\tilde{x}^{k+1}-x^{k}\right\|_{\Omega_{3}^{c}}$ (A16)

$\left\|z^{k+1}-z^{k}\right\|=\left\|D_{\rho}^{1/2}V\left(I-\Sigma^{T}\Sigma_{R}^{-2}\Sigma\right)V^{T}D_{\rho}^{1/2}\left(\hat{x}^{k+1}-x^{k}\right)\right\|\leq\Delta^{k}\left(1+\tau_{\max}^{k}\right)\rho_{\max}\left\|\tilde{x}^{k+1}-x^{k}\right\|_{\Omega_{3}^{c}}$ (A17)

Inequalities (A15), (A16), and (A17) show that the nodal updates are all bound by finite multiples of$\Delta^{k}\left\|\tilde{x}^{k+1}-x^{k}\right\|_{\Omega_{3}^{c}}$. Note that $\lim_{k\to\infty} \Delta^{k}=0$ for the series $\Delta^{k+1}=\Delta^{k}-a\left(\Delta^{k}\right)^{2}$.

**Part II: Convergence of the surrogate function**

In this section, we will show that in the consecutive updates in *x*- (Rule 3) and *y*-optimizations (Rule 5), *z*- (Rule 5), and *x*-updates (Rule 6), the convex surrogate function converges uniformly.

## **The convergence of the surrogate function H to W**

$G\left(x^{k+1},y,^{k+1},z^{k+1}\right)-G\left(x^{k},y^{k},z^{k}\right)=0$. For, the change in *H* becomes:

$H\left(x^{k+1},y,^{k+1},z^{k+1}\right)-H\left(x^{k},y^{k},z^{k}\right)=U\left(x^{k+1}\right)-U\left(x^{k}\right)$ (A18)

The strong convexity of *U* in [*x^k^*, *x^k+1^*] yields:

$\begin{matrix} U\left(x^{k+1}\right)\leq U\left(x^{k}\right)+\Delta^{k}\left[U\left(\hat{\zeta}^{k+1}+\hat{\varepsilon}^{k+1}\right)-U\left(x^{k}\right)\right]-\frac{\Delta^{k}\left(1-\Delta^{k}\right)c_{U}}{2}\left\|\hat{\zeta}^{k+1}+\hat{\varepsilon}^{k+1}-x^{k}\right\|^{2} \\ =U\left(x^{k}\right)+\Delta^{k}\left[U\left(\hat{\zeta}^{k+1}\right)-U\left(x^{k}\right)\right]-\frac{\Delta^{k}\left(1-\Delta^{k}\right)c_{U}}{2}\left\|\hat{\zeta}^{k+1}-x^{k}\right\|^{2}+\vartheta\left(\Delta^{k}\tau_{\max}^{k}\right) \end{matrix}$ (A19)

and that in [$x^{k}, \hat{\zeta}^{k+1}$] yields:

$U\left(\hat{\zeta}^{k+1}\right)-U\left(x^{k}\right)\leq\left[\nabla_{x}U\left(\hat{\zeta}^{k+1}\right)\right]^{T}\left(\hat{\zeta}^{k+1}-x^{k}\right)-\frac{c_{U}}{2}\left\|\hat{\zeta}^{k+1}-x^{k}\right\|^{2}$ (A20)

Inequalities (A19) and (A20) yield:

$U\left(x^{k+1}\right)\leq U\left(x^{k}\right)+\Delta^{k}\left[\nabla_{x}U\left(\hat{\zeta}^{k+1}\right)\right]^{T}\left(\hat{\zeta}^{k+1}-x^{k}\right)-\Delta^{k}c_{U}\left(1-\frac{\Delta^{k}}{2}\right)\left\|\hat{\zeta}^{k+1}-x^{k}\right\|^{2}+\vartheta\left(\tau_{\max}^{k}\right)$ (A21)

The Lipschitz continuity of $\nabla_{x}U\left(x\right)$ yields $\left[\nabla_{x}U\left(\hat{\zeta}^{k+1}\right)-\nabla_{x}U\left(x^{k}\right)\right]^{T}\left(\hat{\zeta}^{k+1}-x^{k}\right)\leq L_{\nabla U}\left\|\hat{\zeta}^{k+1}-x^{k}\right\|^{2}$, and Inequality (A21) leads to:

$U\left(x^{k+1}\right)\leq U\left(x^{k}\right)+\Delta^{k}\left[\nabla_{x}U\left(x^{k}\right)\right]^{T}\left(\hat{\zeta}^{k+1}-x^{k}\right)-c_{U}\Delta^{k}\left(1-\frac{\Delta^{k}}{2}-\frac{L_{\nabla U}}{c_{U}}\right)\left\|\hat{\zeta}^{k+1}-x^{k}\right\|^{2}+\vartheta\left(\tau_{\max}^{k}\right)$ (A22)

Using Eq. (A6), the second term in Inequality (A22) is written as follows:

$\left[\nabla_{x}U\left(x^{k}\right)\right]^{T}\left(\hat{\zeta}^{k+1}-x^{k}\right)=\left[\nabla_{x}U\left(x^{k}\right)\right]^{T}\Phi^{T}\left(\Phi D_{\rho}\Phi^{T}\right)^{-1}\Phi D_{\rho}\left(\tilde{x}^{k+1}-x^{k}\right)_{\Omega_{3}^{c}}$ (A23)

Using Eq. (A3) and *Фz^k^* = 0, we arrive at:

$\begin{matrix} \left[\nabla_{x}U\left(\tilde{x}^{k+1}\right)\right]^{T}\Phi^{T}\left(\Phi D_{\rho}\Phi^{T}\right)^{-1}\Phi D_{\rho}\left(\tilde{x}^{k+1}-x^{k}\right)_{\Omega_{3}^{c}}=-\left(\tilde{x}^{k+1}-x^{k+1}\right)^{T}D_{\rho}\Phi^{T}\left(\Phi D_{\rho}\Phi^{T}\right)^{-1}\Phi D_{\rho}\left(\tilde{x}^{k+1}-x^{k}\right)_{\Omega_{3}^{c}} \\ =-\left(\tilde{x}^{k+1}-x^{k}\right)^{T}D_{\rho}\Phi^{T}\left(\Phi D_{\rho}\Phi^{T}\right)^{-1}\Phi D_{\rho}\left(\tilde{x}^{k+1}-x^{k}\right)_{\Omega_{3}^{c}}+\left(x^{k+1}-x^{k}\right)^{T}D_{\rho}\Phi^{T}\left(\Phi D_{\rho}\Phi^{T}\right)^{-1}\Phi D_{\rho}\left(\tilde{x}^{k+1}-x^{k}\right)_{\Omega_{3}^{c}} \\ =-\left\|V_{R}^{T}D_{\rho}^{1/2}\left(\tilde{x}^{k+1}-x^{k}\right)\right\|_{\Omega_{3}^{c}}^{2}+\left(y^{k+1}-y^{k}\right)^{T}\Phi D_{\rho}\left(\tilde{x}^{k+1}-x^{k}\right)_{\Omega_{3}^{c}}=-\left\|V_{R}^{T}D_{\rho}^{1/2}\left(\tilde{x}^{k+1}-x^{k}\right)\right\|_{\Omega_{3}^{c}}^{2}+\left(x^{k+1}-x^{k}\right)^{T}D_{\rho}\left(\tilde{x}^{k+1}-x^{k}\right)_{\Omega_{3}^{c}} \\ =-\left\|V_{R}^{T}D_{\rho}^{1/2}\left(\tilde{x}^{k+1}-x^{k}\right)\right\|_{\Omega_{3}^{c}}^{2}+\left\|D_{\rho}^{1/2}\left(x^{k+1}-x^{k}\right)\right\|^{2} \end{matrix}$ (A24)

Ineq. (A15) and Eq. (A24) yield:

$\left[\nabla_{x}U\left(\tilde{x}^{k+1}\right)\right]^{T}\Phi^{T}\left(\Phi D_{\rho}\Phi^{T}\right)^{-1}\Phi D_{\rho}\left(\tilde{x}^{k+1}-x^{k}\right)_{\Omega_{3}^{c}}\leq-\rho_{\min}\left\|\tilde{x}^{k+1}-x^{k}\right\|_{\Omega_{3}^{c}}^{2}+\left(\Delta^{k}\right)^{2}\rho_{\max}\left\|\tilde{x}^{k+1}-x^{k}\right\|_{\Omega_{3}^{c}}^{2}$ (A25)

By the definition of $\hat{\zeta}^{k+1}$, it follows that:

$\left\|\hat{\zeta}^{k+1}-x^{k}\right\|=\left\|\Phi^{T}\left(\Phi D_{\rho}\Phi^{T}\right)^{-1}\Phi D_{\rho}\left(\tilde{x}^{k+1}-x^{k}\right)\right\|_{\Omega_{3}^{c}} \leq\sqrt{\frac{\rho_{\max}}{\rho_{\min}}}\left\|\tilde{x}^{k+1}-x^{k}\right\|_{\Omega_{3}^{c}}$ (A26)

Ineq. (A22) becomes:

$U\left(x^{k+1}\right)\leq U\left(x^{k}\right)-\Delta^{k}\left[\rho_{\min}+\frac{\rho_{\max}}{\rho_{\min}}c_{U}-\left(\frac{\Delta^{k}}{2}c_{U}+L_{\nabla U}\right)\frac{\rho_{\max}}{\rho_{\min}}-\left(\Delta^{k}\right)^{2}\rho_{\max}\right]\left\|\tilde{x}^{k+1}-x^{k}\right\|_{\Omega_{3}^{c}}^{2}+\vartheta\left(\tau_{\max}^{k}\right)$ (A27)

For a given *Δ^k^* in (0, *Δ_max_*] and a given ratio *ρ_min_/ρ_max_*, there exists $\rho_{\min}^{cr}$ such that $\rho_{\min}^{cr}=\max\left\{0,\frac{L_{\nabla U}-\left(1-\frac{\Delta_{\max}}{2}\right)c_{U}}{\frac{\rho_{\min}}{\rho_{\max}}-\Delta_{\max}^{2}}\right\}$. From Rule 5 in the proposed algorithm, $\Delta^{k+1}=-a\left(\Delta^{k}-\frac{1}{2a}\right)^{2}+\frac{1}{4a}$, which leads to $\Delta^{k+1}\leq\frac{1}{4a}$, and the equality holds when $\Delta^{k}=\frac{1}{2a}$. As *k* increases, Rule 5 ensures that *Δ^k^* decreases monotonously. If *a* is set at less than ¼, $\rho_{\min}^{cr}$ is strictly positive. Since the ADMM-type constraint function *G* does not change at the iteration, we have the following:

$H\left(x^{k+1},y^{k+1},z^{k+1}\right)\leq H\left(x^{k},y^{k},z^{k}\right)-\Delta^{k}\left[\rho_{\min}+\frac{\rho_{\max}}{\rho_{\min}}c_{U}-\left(\frac{\Delta^{k}}{2}c_{U}+L_{\nabla U}\right)\frac{\rho_{\max}}{\rho_{\min}}-\left(\Delta^{k}\right)^{2}\rho_{\max}\right]\left\|\tilde{x}^{k+1}-x^{k}\right\|_{\Omega_{3}^{c}}^{2}+\vartheta\left(\tau_{\max}^{k}\right)$ (A28)

For a sufficiently large *ρ* > $\rho_{\min}^{cr}$(> 0), the objective function *H*‘s surrogate function always decreases with each iteration. Because *H* is coercive and always improves at iteration, the surrogate function converges.

**Part III: Convergence of the proposed algorithm**

In this section, we present the proof of the convergence of the proposed algorithm by showing: 1) the series of solutions to the surrogate function found through iteration is Lipchitz continuous; 2) the surrogate function and the nonconvex OPF share a fixed point; and 3) the proposed algorithm converges to the shared fixed point, which is a solution to the nonconvex OPF.

## **Lipschitz continuity of $\tilde{\chi}_{j}^{k+1}\left(\chi_{j}^{k}\right)$ and the fixed point**

Let *χ_j_* be the aggregated nodal variable (*x_j_*, *y*, *z_j_*). For an initial point χ^0^, $\tilde{x}_{j}\left(\chi^{0}\right)\triangleq\mathop{\mathrm{argmin}}\limits_{x_{j}} h_{j}\left(\left.x_{j}\right|\chi^{0}\right)$ is uniquely defined. All $\chi_{j}^{0}$ share the global voltage *y* only and are otherwise independent, which makes that . Since *x_j_*, *y*, and *z_j_* are all determined by $\tilde{x}_{j}$, $\tilde{\chi}_{j}$ is the unique solution to . For the unique solution $\tilde{\chi}_{j}$ and a convex function *h_j_*, where $h_{j}\left[\tilde{\chi}_{j}\left(\chi_{j}^{0}\right)\right]\leq h_{j}\left(\chi_{j}^{0}\right)$, the optimality condition provides the following result:

$\left\{\nabla_{\chi_{j}}h_{j}\left[\tilde{\chi}_{j}\left(\chi_{j}^{0}\right)\right]\right\}^{T}\left[\chi_{j}^{0}-\tilde{\chi}_{j}\left(\chi_{j}^{0}\right)\right]\geq0$ (A29)

By the definition of *h_j_* in Eq. (A2), the optimality condition is:

$\left\{\nabla_{\chi_{j}}h_{j}\left[\tilde{\chi}_{j}\left(\chi_{j}^{0}\right)\right]\right\}^{T}\left[\chi_{j}^{0}-\tilde{\chi}_{j}\left(\chi_{j}^{0}\right)\right]=\left\{\nabla_{\chi_{j}}u_{j}\left[\tilde{\chi}_{j}\left(\chi_{j}^{0}\right)\right]+\nabla_{\chi_{j}}g_{j}\left[\tilde{\chi}_{j}\left(\chi_{j}^{0}\right)\right]\right\}^{T}\left[\chi_{j}^{0}-\tilde{\chi}_{j}\left(\chi_{j}^{0}\right)\right]\geq0$ (A30)

The convexity of *g_j_* yields:

$g_{j}\left(\chi_{j}^{0}\right)\geq g_{j}\left[\tilde{\chi}_{j}\left(\chi_{j}^{0}\right)\right]+\left\{\nabla_{\chi_{j}}g_{j}\left[\tilde{\chi}_{j}\left(\chi_{j}^{0}\right)\right]\right\}^{T}\left[\chi_{j}^{0}-\tilde{\chi}_{j}\left(\chi_{j}^{0}\right)\right]+\frac{c_{g}}{2}\left\|\chi_{j}^{0}-\tilde{\chi}_{j}\left(\chi_{j}^{0}\right)\right\|^{2}$ (A31)

Ineq. (A30) becomes

$\left\{\nabla_{\chi_{j}}u_{j}\left[\tilde{\chi}_{j}\left(\chi_{j}^{0}\right)\right]\right\}^{T}\left[\chi_{j}^{0}-\tilde{\chi}_{j}\left(\chi_{j}^{0}\right)\right]+g_{j}\left(\chi_{j}^{0}\right)-g_{j}\left[\tilde{\chi}_{j}\left(\chi_{j}^{0}\right)\right]-\frac{c_{g}}{2}\left\|\chi_{j}^{0}-\tilde{\chi}_{j}\left(\chi_{j}^{0}\right)\right\|^{2}\geq\left\{\nabla_{\chi_{j}}h_{j}\left[\tilde{\chi}_{j}\left(\chi_{j}^{0}\right)\right]\right\}^{T}\left[\chi_{j}^{0}-\tilde{\chi}_{j}\left(\chi_{j}^{0}\right)\right]\geq0$ $\to\left\{\nabla_{\chi_{j}}u_{j}\left[\tilde{\chi}_{j}\left(\chi_{j}^{0}\right)\right]\right\}^{T}\left[\chi_{j}^{0}-\tilde{\chi}_{j}\left(\chi_{j}^{0}\right)\right]+g_{j}\left(\chi_{j}^{0}\right)-g_{j}\left[\tilde{\chi}_{j}\left(\chi_{j}^{0}\right)\right]\geq\frac{c_{g}}{2}\left\|\chi_{j}^{0}-\tilde{\chi}_{j}\left(\chi_{j}^{0}\right)\right\|^{2}$ (A32)

Note that $\nabla_{\chi_{j}}u_{j}\left[\tilde{\chi}_{j}\left(\chi_{j}^{0}\right)\right]=\nabla_{\chi_{j}}f_{j}\left[\tilde{\chi}_{j}\left(\chi_{j}^{0}\right)\right]$, which implies that $\tilde{\chi}_{j}\left(\chi_{j}^{0}\right)$ satisfies the optimality condition for the exact and nonconvex OPF as well. Consider two starting points $\chi^{I}$ and $\chi^{II}$:

$\begin{matrix} \left\{\nabla_{\chi_{j}}u_{j}\left[\tilde{\chi}_{j}\left(\chi_{j}^{I}\right)\right]\right\}^{T}\left[\chi_{j}^{0}-\tilde{\chi}_{j}\left(\chi_{j}^{I}\right)\right]+g_{j}\left(\chi_{j}^{0}\right)-g_{j}\left[\tilde{\chi}_{j}\left(\chi_{j}^{I}\right)\right]\geq0 \\ \left\{\nabla_{\chi_{j}}u_{j}\left[\tilde{\chi}_{j}\left(\chi_{j}^{II}\right)\right]\right\}^{T}\left[\chi_{j}^{0}-\tilde{\chi}_{j}\left(\chi_{j}^{II}\right)\right]+g_{j}\left(\chi_{j}^{0}\right)-g_{j}\left[\tilde{\chi}_{j}\left(\chi_{j}^{II}\right)\right]\geq0 \end{matrix}$ (A33)

The inequalities hold for any feasible $\chi_{j}^{0}$. Set $\chi_{j}^{0}$ to $\tilde{\chi}_{j}\left(\chi_{j}^{II}\right)$ (top) and to $\tilde{\chi}_{j}\left(\chi_{j}^{I}\right)$ (bottom), and add inequalities:

$\left\{\nabla_{\chi_{j}}u_{j}\left[\tilde{\chi}_{j}\left(\chi_{j}^{II}\right)\right]-\nabla_{\chi_{j}}u_{j}\left[\tilde{\chi}_{j}\left(\chi_{j}^{I}\right)\right]\right\}^{T}\left[\tilde{\chi}_{j}\left(\chi_{j}^{II}\right)-\tilde{\chi}_{j}\left(\chi_{j}^{I}\right)\right]\leq0$ (A34)

The function is Lipschitz continuous because *u_j_* is continuously differentiable, i.e., $\left\|\nabla_{\chi_{j}}u_{j}\left[\tilde{\chi}_{j}\left(\chi_{j}^{II}\right)\right]-\nabla_{\chi_{j}}u_{j}\left[\tilde{\chi}_{j}\left(\chi_{j}^{I}\right)\right]\right\|=\left\|u_{j}^{\nabla}\left(\chi_{j}^{II}\right)-u_{j}^{\nabla}\left(\chi_{j}^{I}\right)\right\|\leq L_{u_{j}^{\nabla}}\left\|\chi_{j}^{II}-\chi_{j}^{I}\right\|$. The upper bound of Ineq. (A34) is:

$\begin{matrix} \left\{\nabla_{\chi_{j}}u_{j}\left[\tilde{\chi}_{j}\left(\chi_{j}^{II}\right)\right]-\nabla_{\chi_{j}}u_{j}\left[\tilde{\chi}_{j}\left(\chi_{j}^{I}\right)\right]\right\}^{T}\left[\tilde{\chi}_{j}\left(\chi_{j}^{II}\right)-\tilde{\chi}_{j}\left(\chi_{j}^{I}\right)\right] \\ \leq\left\|\nabla_{\chi_{j}}u_{j}\left[\tilde{\chi}_{j}\left(\chi_{j}^{II}\right)\right]-\nabla_{\chi_{j}}u_{j}\left[\tilde{\chi}_{j}\left(\chi_{j}^{I}\right)\right]\right\|\left\|\tilde{\chi}_{j}\left(\chi_{j}^{II}\right)-\tilde{\chi}_{j}\left(\chi_{j}^{I}\right)\right\|\leq L_{u_{j}^{\nabla}}\left\|\chi_{j}^{II}-\chi_{j}^{I}\right\|\left\|\tilde{\chi}_{j}\left(\chi_{j}^{II}\right)-\tilde{\chi}_{j}\left(\chi_{j}^{I}\right)\right\| \end{matrix}$ (A35)

On the other hand, the strong convexity of $u_{j}\left(\chi_{j}\right)$ yields

$\left\{\nabla_{\chi_{j}}u_{j}\left[\tilde{\chi}_{j}\left(\chi_{j}^{II}\right)\right]-\nabla_{\chi_{j}}u_{j}\left[\tilde{\chi}_{j}\left(\chi_{j}^{I}\right)\right]\right\}^{T}\left[\tilde{\chi}_{j}\left(\chi_{j}^{II}\right)-\tilde{\chi}_{j}\left(\chi_{j}^{I}\right)\right]\geq c_{u}\left\|\tilde{\chi}_{j}\left(\chi_{j}^{II}\right)-\tilde{\chi}_{j}\left(\chi_{j}^{I}\right)\right\|^{2}$ (A36)

Combining Ineq. (A35) and (A36) yields:

$\left\|\tilde{\chi}_{j}\left(\chi_{j}^{II}\right)-\tilde{\chi}_{j}\left(\chi_{j}^{I}\right)\right\|\leq\frac{L_{u_{j}^{\nabla}}}{c_{u}}\left\|\chi_{j}^{II}-\chi_{j}^{I}\right\|$ (A37)

The inequality proves the Lipschitz continuity of $\tilde{\chi}_{j}^{k+1}\left(\chi_{j}^{k}\right)$, i.e., the neighborhood of initial points stays close in the mapping of the function $\tilde{\chi}_{j}^{k+1}\left(\chi_{j}^{k}\right)$. Ineq. (A37) holds for the fixed point $\chi_{j}^{*}$, where $\tilde{\chi}_{j}^{*}\left(\chi_{j}^{*}\right)=\chi_{j}^{*}$: $\left[\nabla_{\chi_{j}}u_{j}\left(\chi_{j}^{*}\right)\right]^{T}\left(\chi_{j}^{0}-\chi_{j}^{*}\right)+g_{j}\left(\chi_{j}^{0}\right)-g_{j}\left(\chi_{j}^{*}\right)\geq0$. For some $g_{j}^{\nabla}\in\partial g_{j}\left(\chi^{*}\right)$, the optimality condition reduces to: $\left[\nabla_{\chi_{j}}u_{j}\left(\chi_{j}^{*}\right)+g_{j}^{\nabla}\right]^{T}\left(\chi_{j}^{0}-\chi_{j}^{*}\right)\geq0$. Summing over all *j* yields $\left[\nabla_{\chi}U\left(\chi^{*}\right)+G_{j}^{\nabla}\right]^{T}\left(\chi^{0}-\chi^{*}\right)\geq0$. Note that $\nabla_{\chi}U\left(\chi\right)=\nabla_{\chi}F\left(\chi\right)$, which implies that *χ^*^* is a stationary point of the nonconvex OPF problem as well. *χ^*^* is the unique solution to the SDP with initial point of *χ^*^*, and *χ^*^* satisfies the optimality conditions. Therefore, the nonconvex OPF and its relaxed SDP share a fixed point.

## **Convergence of the nonconvex OPF**

For the *k*^th^ iteration and an initial point $\chi_{j}^{0}=\chi_{j}^{k}$, Ineq. (A36) leads to:

$\left\{\nabla_{\chi_{j}}u_{j}\left[\tilde{\chi}_{j}\left(\chi_{j}^{k}\right)\right]\right\}^{T}\left[\chi_{j}^{k}-\tilde{\chi}_{j}\left(\chi_{j}^{k}\right)\right]+g_{j}\left(\chi_{j}^{k}\right)-g_{j}\left[\tilde{\chi}_{j}\left(\chi_{j}^{k}\right)\right]$ $\geq\frac{c_{g}}{2}\left\|\chi_{j}^{k}-\tilde{\chi}_{j}\left(\chi_{j}^{k}\right)\right\|^{2}$. By $\nabla_{\chi_{j}}f_{j}\left[\tilde{\chi}_{j}\left(\chi_{j}^{k}\right)\right]=\nabla_{\chi_{j}}u_{j}\left[\tilde{\chi}_{j}\left(\chi_{j}^{k}\right)\right]$, $\left\{\nabla_{\chi_{j}}f_{j}\left[\tilde{\chi}_{j}\left(\chi_{j}^{k}\right)\right]\right\}^{T}\left[\chi_{j}^{k}-\tilde{\chi}_{j}\left(\chi_{j}^{k}\right)\right]+g_{j}\left(\chi_{j}^{k}\right)-g_{j}\left[\tilde{\chi}_{j}\left(\chi_{j}^{k}\right)\right]$ $\geq\frac{c_{g}}{2}\left\|\chi_{j}^{k}-\tilde{\chi}_{j}\left(\chi_{j}^{k}\right)\right\|^{2}$. Summing over all *j* gives:

$\left\{\nabla_{\chi}F\left[\tilde{\chi}\left(\chi^{k}\right)\right]\right\}^{T}\left[\chi^{k}-\tilde{\chi}\left(\chi^{k}\right)\right]+\sum_{j} \left\{g_{j}\left(\chi_{j}^{k}\right)-g_{j}\left[\tilde{\chi}_{j}\left(\chi_{j}^{k}\right)\right]\right\}\geq\frac{c_{g}^{\min}}{2}\left\|\chi^{k}-\tilde{\chi}\left(\chi^{k}\right)\right\|^{2}$ (A38)

Since *x_j_*, *y*, and *z_j_* are all determined from $\tilde{x}_{j}$, $\tilde{\chi}_{j}$ is well defined in terms of $\tilde{x}_{j}$. At given *y^k^* and *z^k^*,

$\left\{\nabla_{\chi}F\left[\chi\left(x^{k+1}\right)\right]\right\}^{T}\left[\chi\left(x^{k}\right)-\chi\left(x^{k+1}\right)\right]+\sum_{j} \left\{g_{j}\left[\chi_{j}\left(x^{k}\right)\right]-g_{j}\left[\chi_{j}\left(x^{k+1}\right)\right]\right\}\geq\frac{c_{g}^{\min}}{2}\left\|\chi\left(x^{k}\right)-\chi\left(x^{k+1}\right)\right\|^{2}$ (A39)

Note that *F* is a function of *x* only and that *χ_j_* is (*x_j_*, *y*, *z_j_*). Ineq. (A39) becomes:

$\left[\nabla_{x}F\left(x^{k+1}\right)\right]^{T}\left(x^{k}-x^{k+1}\right)+\sum_{j} \left[g_{j}\left(x^{k},y^{k},z^{k}\right)-g_{j}\left(x^{k+1},y^{k+1},z^{k+1}\right)\right]\geq\frac{c_{g}^{\min}}{2}\left\|\left(\begin{matrix} x^{k}-x^{k+1} \\ y^{k}-y^{k+1} \\ z^{k}-z^{k+1} \end{matrix}\right)\right\|^{2}$ (A40)

Using Ineqs. (A15), (A16), (A17), and $\left\|I-D_{\rho}^{1/2}\Phi^{T}\left(\Phi D_{\rho}\Phi^{T}\right)^{-1}\Phi D_{\rho}^{1/2}\right\|=1$, it can be seen that:

$\left\|\chi\left(x^{k}\right)-\chi\left(x^{k+1}\right)\right\|^{2}\leq\left[\left\|D_{\rho}^{-1}\right\|\left\|D_{\rho}\right\|+\left\|\left(\Phi D_{\rho}\Phi^{T}\right)^{-1}\Phi D_{\rho}\right\|^{2}+\left\|D_{\rho}\right\|\right]\left\|\hat{x}^{k+1}-x^{k}\right\|^{2}$ (A41)

Using $\hat{x}^{k+1}-x^{k}=\Delta^{k}\left(I-\Lambda_{3}\right)\left(\tilde{x}^{k+1}-x^{k}\right)+\Delta^{k}\Lambda_{2}\tilde{b}^{k+1}$,

$\left\|\hat{x}^{k+1}-x^{k}\right\|\leq\Delta^{k}\left\|\tilde{x}^{k+1}-x^{k}\right\|_{\Omega_{3}^{c}}+\Delta^{k}\tau_{\max}^{k}\left\|\tilde{x}^{k+1}-x^{k}\right\|_{\Omega_{2}}\leq\Delta^{k}\left(1+\tau_{\max}^{k}\right)\left\|\tilde{x}^{k+1}-x^{k}\right\|_{\Omega_{3}^{c}}$ (A42)

Ineq. (A40) becomes:

$\left[\nabla_{x}F\left(x^{k+1}\right)\right]^{T}\left(x^{k}-x^{k+1}\right)+\sum_{j} \left[g_{j}\left(x^{k},y^{k},z^{k}\right)-g_{j}\left(x^{k+1},y^{k+1},z^{k+1}\right)\right]\geq\frac{c_{g}^{\min}}{2}C_{W}^{k+1}\left(\Delta^{k}\right)^{2}\left(1+\tau_{\max}^{k}\right)^{2}\left\|\tilde{x}^{k+1}-x^{k}\right\|_{\Omega_{3}^{c}}^{2}$ (A43)

where $C_{W}^{k+1}=\left\|D_{\rho}^{-1}\right\|\left\|D_{\rho}\right\|+\left\|\left(\Phi D_{\rho}\Phi^{T}\right)^{-1}\Phi D_{\rho}\right\|^{2}+\left\|D_{\rho}\right\|$.

Since *U* is strongly convex, $\left[\nabla_{x}U\left(x^{k}\right)\right]^{T}$ $\times\left(x^{k}-x^{k+1}\right)-\frac{c_{U}}{2}\left\|x^{k+1}-x^{k}\right\|^{2}\geq\left[\nabla_{x}U\left(x^{k+1}\right)\right]^{T}\left(x^{k}-x^{k+1}\right)$. Ineq. (A43) becomes:

$\left[\nabla_{x}F\left(x^{k}\right)\right]^{T}\left(x^{k+1}-x^{k}\right)+\sum_{j} \left[g_{j}\left(x^{k+1},y^{k+1},z^{k+1}\right)-g_{j}\left(x^{k},y^{k},z^{k}\right)\right]\leq-\frac{c_{g}^{\min}C_{W}^{k+1}+c_{U}\left\|D_{\rho}^{-1}\right\|\left\|D_{\rho}\right\|}{2}\left(\Delta^{k}\right)^{2}\left(1+\tau_{\max}^{k}\right)^{2}\left\|\tilde{x}^{k+1}-x^{k}\right\|_{\Omega_{3}^{c}}^{2}$(A44)

For the *k*^th^ iteration, the Descent Lemma results in [1]:

$\begin{matrix} F\left(x^{k+1}\right)\leq F\left(x^{k}\right)+\nabla_{x}F\left(x^{k}\right)^{T}\left(x^{k+1}-x^{k}\right)+\frac{L_{F}}{2}\left\|x^{k+1}-x^{k}\right\|^{2} \\ \leq F\left(x^{k}\right)+\nabla_{x}F\left(x^{k}\right)^{T}\left(x^{k+1}-x^{k}\right)+\frac{L_{F}}{2}\left(\Delta^{k}\right)^{2}\left(1+\tau_{\max}\right)^{2}\left\|D_{\rho}^{-1}\right\|\left\|D_{\rho}\right\|\left\|\tilde{x}^{k+1}-x^{k}\right\|_{\Omega_{3}^{c}}^{2} \end{matrix}$ (A45)

Combining (A44), and (A45) yields:

$$\begin{matrix} W\left(\chi^{k+1}\right)=F\left(x^{k+1}\right)+\sum_{j} g_{j}\left(\chi^{k+1}\right) \\ \leq F\left(x^{k}\right)+\sum_{j} g_{j}\left(\chi^{k}\right)+\nabla_{x}F\left(x^{k}\right)^{T}\left(x^{k+1}-x^{k}\right)+\sum_{j} g_{j}\left(\chi^{k+1}\right)-\sum_{j} g_{j}\left(\chi^{k}\right) +\frac{L_{F}}{2}\left(\Delta^{k}\right)^{2}\left(1+\tau_{\max}^{k}\right)^{2}\left\|D_{\rho}^{-1}\right\|\left\|D_{\rho}\right\|\left\|\tilde{x}^{k+1}-x^{k}\right\|_{\Omega_{3}^{c}}^{2} \\ \leq W\left(\chi^{k}\right)-\eta_{w}^{k+1}\left(\Delta^{k}\right)^{2}\left\|\tilde{x}^{k+1}-x^{k}\right\|_{\Omega_{3}^{c}}^{2}+\tau_{\max}^{k}\left[-\eta_{w}^{k+1}\left(2+\tau_{\max}\right)\left(\Delta^{k}\right)^{2}\right]\left\|\tilde{x}^{k+1}-x^{k}\right\|_{\Omega_{3}^{c}}^{2} \end{matrix}$$

where $\eta_{w}^{k+1}=\frac{c_{g}^{\min}C_{W}^{k+1}+\left\|D_{\rho}^{-1}\right\|\left\|D_{\rho}\right\|\left(c_{U}-L_{F}\right)}{2}$ (A46)

The sequence $\tau_{\max}^{k}\left[-\eta_{w}^{k+1}\left(2+\tau_{\max}^{k}\right)\left(\Delta^{k}\right)^{2}\right]\left\|\tilde{x}^{k+1}-x^{k}\right\|_{\Omega_{3}^{c}}^{2}$ is bounded by $\tau_{\max}\left[-\eta_{w}^{k+1}\left(2+\tau_{\max}^{k}\right)\left(\Delta^{k}\right)^{2}\right]\left\|\varepsilon^{k}\right\|^{2}$. It is noted that $\sum_{k=1}^{\infty} \left\|\varepsilon^{k}\right\|^{2}\leq\left\|\tilde{x}_{j}^{k+1}-x_{j}^{k}\right\|_{\max}^{2}\sum_{k=1}^{\infty} \left(\tau_{\max}^{k}\right)^{2}<\infty$ for a sequence $\tau_{\max}^{k}=\tau_{\max}^{0}/k.$ Since $\sum_{k=1}^{\infty} \left\|\varepsilon^{k}\right\|^{2}<\infty$, the sum of the sequence $\tau_{\max}\left[-\eta_{w}^{k+1}\left(2+\tau_{\max}^{k}\right)\left(\Delta^{k}\right)^{2}\right]\left\|\tilde{x}^{k+1}-x^{k}\right\|_{\Omega_{3}^{c}}^{2}$ is finite.

Using the Lemma 3.4 in [2], either *W*(*χ^k^*) divulges to -∞ or *W*(*χ^k^*) converges to a finite value and $\sum_{k=1}^{\infty} \eta_{w}^{k+1}\left(\Delta^{k}\right)^{2}\left\|\tilde{x}^{k+1}-x^{k}\right\|_{\Omega_{3}^{c}}^{2}<\infty$. *W* is coercive, which implies that *W*(*χ^k^*) does not divulge. Therefore, *W*(*χ^k^*) converges to a finite value, and $\sum_{k=1}^{\infty} \eta_{w}^{k+1}\left(\Delta^{k}\right)^{2}\left\|\tilde{x}^{k+1}-x^{k}\right\|_{\Omega_{3}^{c}}^{2}<\infty$. It is proven that $\lim_{k\to\infty} \left\|\tilde{x}^{k+1}-x^{k}\right\|_{\Omega_{3}^{c}}^{2}=0$ in [24], which means that the sequence {*χ^k^*}, using an inexact search, finds a local solution to the nonconvex OPF.

**References**

1. Bertsekas DP, Tsitsiklis JN. Parallel and Distributed Computation: Numerical Methods. Parallel and Distributed Computation Numerical Methods. 1989.

2. D. P. Bertsekas and J. N. Tsitsiklis. Neuro-dynamic programming. Cambridge, Massachusetts: Athena Scientific Press; 2011.
